# Nrf-2 signaling inhibits intracranial aneurysm formation and progression by modulating vascular smooth muscle cell phenotype and function

**DOI:** 10.1186/s12974-019-1568-3

**Published:** 2019-10-04

**Authors:** Yuan Shi, Sichen Li, Yaying Song, Peixi Liu, Zixiao Yang, Yingjun Liu, Kai Quan, Guo Yu, Zhiyuan Fan, Wei Zhu

**Affiliations:** 1 Department of Neurosurgery, Huashan Hospital, Fudan University, 12 Wulumiqi Rd., Shanghai, 200040 People’s Republic of China; 2 Department of Neurology, Ruijin Hospital, Shanghai Jiao Tong University School of Medicine, 197 Ruijin Rd. No.2, Shanghai, 200025 China

**Keywords:** Intracranial aneurysm, Nrf-2, Vascular smooth muscle cells phenotype, Oxidative stress, Inflammation

## Abstract

**Background:**

Oxidative stress and vascular smooth muscle cell (VSMC) phenotypic modulation influence intracranial aneurysm (IA) formation and progression. Oxidative stress plays an important role in phenotype switching, and nuclear factor erythroid 2-related factor 2 (Nrf-2) is one of the main antioxidant systems. Unfortunately, little is known about how Nrf-2 signaling influences VSMC phenotype switches during IA pathogenesis.

**Methods:**

We examined the effect of Nrf-2 activation IA on formation and progression in an elastase-induced rat IA model. We also developed a hydrogen peroxide (H_2_O_2_)-induced VSMC oxidative damage model. Then, we analyzed VSMC phenotype changes in the setting of Nrf-2 activation or inhibition in vitro. The proliferation, migration ability, and apoptosis rate of VSMCs were tested. Lastly, we measured the expression levels of antioxidant enzymes and inflammatory cytokines downstream of Nrf-2.

**Results:**

Nrf-2 activation suppressed IA formation and progression in vivo. We confirmed Nrf-2 nuclear translocation and a VSMC switch from the contractile to synthetic phenotype. Nrf-2 activation inhibited the proliferation, migratory ability, and apoptosis rate enhanced by H_2_O_2_. Quantitative real-time polymerase chain reaction (PCR) and western blot analysis revealed that Nrf-2 activation promoted antioxidant enzymes and VSMC-specific marker gene expressions but decreased pro-inflammatory cytokine levels.

**Conclusion:**

These results suggest that Nrf-2 exerts protective effects against IA development by preventing VSMCs from changing to a synthetic phenotype.

## Background

Intracranial aneurysms (IAs) are pathological dilations at main bifurcations of cerebral arteries and are the most common cause of subarachnoid hemorrhage. Nearly 3–5% of the adult population carries unruptured IA [1]. The mechanisms of IA development and rupture remain unclear. Many factors such as blood flow wall shear stress, inflammation, oxidative stress, and apoptosis are involved in IA etiology [2]. The abnormal hemodynamic changes that can lead to endothelial cell dysfunction are now considered to be the initiating factor [3]. Several inflammatory mediators are triggered and then release large amounts of inflammatory cytokines and oxidative factors. This can cause a number of pathological processes including disruption of internal elastic laminate, vascular smooth muscle cell (VSMC) phenotype modulation, and dysfunctional extracellular matrix (ECM) remodeling [2–4]. When vessel walls degenerate, intracranial arteries become too weak to resist the blood dynamic force, and pouch-like dilatations of cerebral arteries occur.

VSMCs are a major cell type in vessel walls that carry out various functions. There are two different types of VSMCs: contractile and synthetic. Markers of contractile phenotype include smooth muscle 22 alpha (SM22α), smooth muscle alpha actin (αSMA), SM myosin heavy chain (MHC), h1-calponin, and smoothelin [5]. In response to pathologic stimuli such as inflammation and oxidative stress, contractile phenotype VSMCs can convert to the synthetic type; this phenotype modulation is associated with deceased expression of contractile genes [6]. During these changes, VSMCs lose their ability to contract but contribute to recruitment of pro-inflammatory cells and remodeling of the vessel wall ECM [6]. Switching to synthetic type VSMCs is hypothesized to play an important role in several cardiovascular diseases [7].

Oxidative stress is a key contributor to IA formation and rupture [8]. Oxidative damage caused by reactive oxygen species (ROS) can injure vessel walls by generating unstable free radicals and recruiting pro-inflammatory cells [5]. ROS also play an essential role in inflammatory disorders and VSMC phenotype modulation [9]. Nuclear factor erythroid 2-related factor 2 (Nrf-2), which belongs to the CNC (cap ‘n’ collar) family of transcription factors, is one of the main endogenous antioxidant systems. In stress conditions, Nrf-2 translocates from the cytoplasm to the nucleus where it regulates the expression of antioxidant and anti-inflammatory genes [10]. However, little is known about the significance of Nrf-2 in IA pathophysiology. In the current study, we tested the hypothesis that activation of Nrf-2 signaling can inhibit IA progression by modulating VSMC phenotype and function.

## Methods

### Patients and tissue samples

The Institutional Review Board of the Huashan Hospital of Fudan University approved this study. Informed consent was obtained from all patients. IA and superficial temporal artery (STA) samples were obtained during surgery. All specimens were fixed in 10% formaldehyde and embedded in paraffin.

### Rat IA model

All animal procedures were carried out according to the protocol of our Institutional Animal Care and Use Committee. The experimental protocol was reviewed and approved by the Ethics Committee of the Huashan Hospital affiliated with Fudan University in Shanghai, People’s Republic of China. Male adult Sprague Dawley rats (body weight 200–220 g; Jiesijie, China) were divided into two groups (*n* = 10). IAs were induced as described previously [11]. Briefly, the right common carotid artery of rats was ligated, and 10 μL of 10 U/mL elastase was stereotactically injected into the basal cisterns through a small burr hole made 1.2 mm rostral and 0.7 mm lateral to the right of the bregma. The rats were anesthetized with 3% isoflurane throughout the procedure. All rats were fed a hypertensive diet after the surgery, and one group was treated with the Nrf-2 agonist tert-butylhydroquinone (tBHQ, MedChemExpress, NJ, USA) 50 mg/kg/d by gavage. After 30 days, rats were perfused with phosphate-buffered saline (PBS) and 4% paraformaldehyde (PFA) under deep anesthesia, then infused with bromophenol blue solution. Aneurysms were defined as an outpouching of weakened vessel walls, the diameters of which were 150% greater than the patent artery. All samples were processed immediately as described above for human specimens.

### Hematoxylin and eosin (HE) staining and immunohistochemistry

Tissues were cut into 5-μM sections and placed on polylysine-coated slides prior to routine HE staining. For immunohistochemical analysis, anti-Nrf-2 (ab28947, Abcam, Cambridge, UK) (ab28947, Abcam, Cambridge, UK) was used for primary antibodies, and 3,3′-diaminobenzidine (DAB) plus chromogen (Thermo Fisher Scientific, Waltham, MA) was used for substrate visualization, according to the manufacturers’ protocols.

### Immunofluorescence staining

Five-micrometer-thick sections were incubated at 4 °C overnight with anti- SM22α (1:400 dilution) and anti-αSMA (1:200 dilution) (Abcam, Cambridge, UK). They were then incubated with appropriate fluorescence-labeled secondary antibodies (1:1000 dilution) for 2 h at room temperature. Nuclei were stained with DAPI (1:1000 dilution). All images were obtained with a fluorescence microscope (Leica Microsystems, Oberkochen, Germany).

### Rat VSMC isolation and culture

Primary rats VSMCs were isolated and cultured using previously described methods [12]. After VSMCs migrated from tissue pieces in Dulbecco’s minimum essential medium (DMEM; Gibco, Grand Island, NY) supplemented with 20% fetal bovine serum (FBS; Gemini, West Sacramento, CA), they were collected and cultured in DMEM supplemented with 10% FBS, 100 U/mL penicillin, and 100 mg/mL streptomycin. Cells were grown in a humidified incubator (Thermo Fisher Scientific) at 37 °C in a 5% CO_2_ atmosphere, and VSMCs at passages 3 to 8 were used for all experiments. VSMCs were treated with hydrogen peroxide (H_2_O_2_, 0.5 mM) for 12 h to induce oxidative damage and pre-treated with tBHQ (20 μM/mL) for 24 h and Nrf-2 inhibitor ML385 (MedChemExpress, NJ, USA) (5 μM/mL) for 72 h [13] before stimulation with H_2_O_2_. VSMCs were harvested for mRNA and protein analyses and functional testing. Each experiment was performed at least three times with triplicate cultures.

### Cell viability and proliferation assay

To choose appropriate concentrations of H_2_O_2_ and tBHQ, cell viability was evaluated by a Cell Counting Kit-8 (CCK-8, Dojindo Molecular Technologies, Gaithersburg, MD) according to the manufacturer’s instruction. Cells were seeded in 96-well plate with 5 × 10^3^ cells/well and were cultured until 80% confluent. After treatment with different dosages of H_2_O_2_ (10, 1, 0.5, 0.2, 0.1 mM) for 12 h and tBHQ (1, 10, 20, 30, 40, 50 μM/mL) for 24 h, 10 μL of CCK-8 solution was added into the culture medium of each well, and then cells were incubated in humidified 95% air and 5% CO_2_ for 2 h at 37 °C. To evaluate the influence of Nrf-2 signaling on proliferation, cell proliferation tests were performed as mentioned above after treatment with tBHQ and ML 385. Absorbance was measured at 450 nm using a microplate reader (Bio-Tek, Winooski, VT).

### Intracellular ROS measurement

To determine intracellular ROS levels, cells were seeded in 6-well plate (1 × 10^5^ cells/well). After treatment as described above, DCFH-DA fluorescence probes (1:1000 dilution; Beyotime, Haimen, China) were loaded, and cells were incubated for 1 h at 37 °C. Then, VSMCs were washed three times with serum-free DMEM and suspended in PBS. The fluorescence intensity of DCF was measured at wavelengths of 488 nm (excitation) and 525 nm (emission) in a flow cytometer (Thermo Fisher Scientific, Waltham, MA).

### Apoptosis analysis

The Dead Cell Apoptosis Kit with Annexin V Alexa & propidium iodide (PI) (Invitrogen, Carlsbad, CA) was employed to measure the apoptosis rate. Cells were treated as mentioned above, and agent-free culture was used as negative control. Next, VSMCs were harvested and washed twice with PBS. Cells were resuspended in 1× annexin-binding buffer and then stained with 5 μL of PI and FITC Annexin-V for 15 min at room temperature in the dark. After the incubation period, 400 μL 1× annexin-binding buffer was added, and stained cells were measured by flow cytometry, analyzing fluorescence emission at 530 nm and excitation at 488 nm.

### Hoechst staining

For staining, cells were seeded in 24-well plates at 2 × 10^4^ cells/well and cultured to 60–70%. After treatment, cells were fixed with fixation buffer for 10 min. After being washed three times with PBS, cells were incubated in 0.2 mL Hoechst (Beyotime) for 5 min and then washed three times with PBS. The images were captured using a fluorescence microscope (Leica) at wavelength of 350 nm (excitation) and 460 nm (emission). The count of positive cells was analyzed by ImageJ software (National Institutes of Health, Bethesda, MD).

### Cell migration assay

For wound healing assays, cells were seeded into 24-well plates, treated as described above, and cultured until 90% confluence. After scratching by a 10-μL pipette tip, the wound width was photographed and measured by ImageJ at 0 and 24 h. For transwell assays, cells suspensions of 5 × 10^4^ cells/mL were seeded in the upper chambers and incubated with Nrf-2 agonist or inhibitor. After treatment with H_2_O_2_ for 12 h, DMEM with 10% FBS was added to the bottom chambers to stimulate migration. Cells in the upper chambers were scraped after 24 h. Migrated cells were fixed with 4% PFA and stained in crystal violet. Numbers of migrated cells were assessed under light microscopy by counting six fields.

### RNA isolation and quantitative real-time polymerase chain reaction (qRT-PCR) analysis

Total RNA was extracted by using TRIzol (Takara, Kusatsu, Japan) according to the manufacturer’s protocol. For qRT-PCR analysis, cDNA was synthesized using ABScript II cDNA First-Strand Synthesis Kit under to manufacturer instructions. mRNA expression was evaluated with SYBR green-based PCR reactions. The results were analyzed using the ΔΔCt method and expressed as ratio of the internal control, glyceraldehyde 3-phosphate dehydrogenase (GAPDH).

### Cytokine and chemokine analysis

Cellular supernatants were collected (centrifuge at 1000 g for 5 min) after cells were treated as mentioned above. We choose interleukin 1 beta (IL-1b), IL-6, monocyte chemoattractant protein 1 (MCP-1), and tumor necrosis factor alpha (TNF-a). Cytokines were measured via a MILLIPLEX MAP Rat CVD Panel (Millipore, Billerica, MA).

### Western blot analysis

The proteins were collected using RIPA lysis buffer. To determine the expression of nuclear transcription factor Nrf-2, nuclear and cytoplasmic proteins were extracted separately. Cell lysates were separated by sodium dodecyl sulfate-polyacrylamide gel electrophoresis and then transferred to polyvinylidene fluoride membranes. After membranes were blocked, proteins were detected with the following primary antibodies (Abcam, Cambridge, UK): anti-Nrf-2 (Ab137550), anti-NAD(P)H quinone dehydrogenase 1 (NQO-1) (ab28947), anti-SM22α (ab14106), and anti-αSMA (ab7817). The membranes were exposed to X-ray after being probed with enhanced chemiluminescence and visualized with an imaging system (Tanon, Shanghai, China). The results were calculated by ImageJ software.

### Statistical analysis

Each experiment was performed at least three times with triplicate groups. Data are the means ± standard errors of the mean. All statistical analyses were performed using one-way analysis of variance in IBM SPSS statistics 23 (IBM Corp., Armonk, NY), and figures were generated in GraphPad Prism software version 7.0 (GraphPad Inc., San Diego, CA). *p* < 0.05 was considered significant.

## Results

### Nrf-2 signaling can inhibit IA formation and progression in a rat IA model

**Fig. 1:**
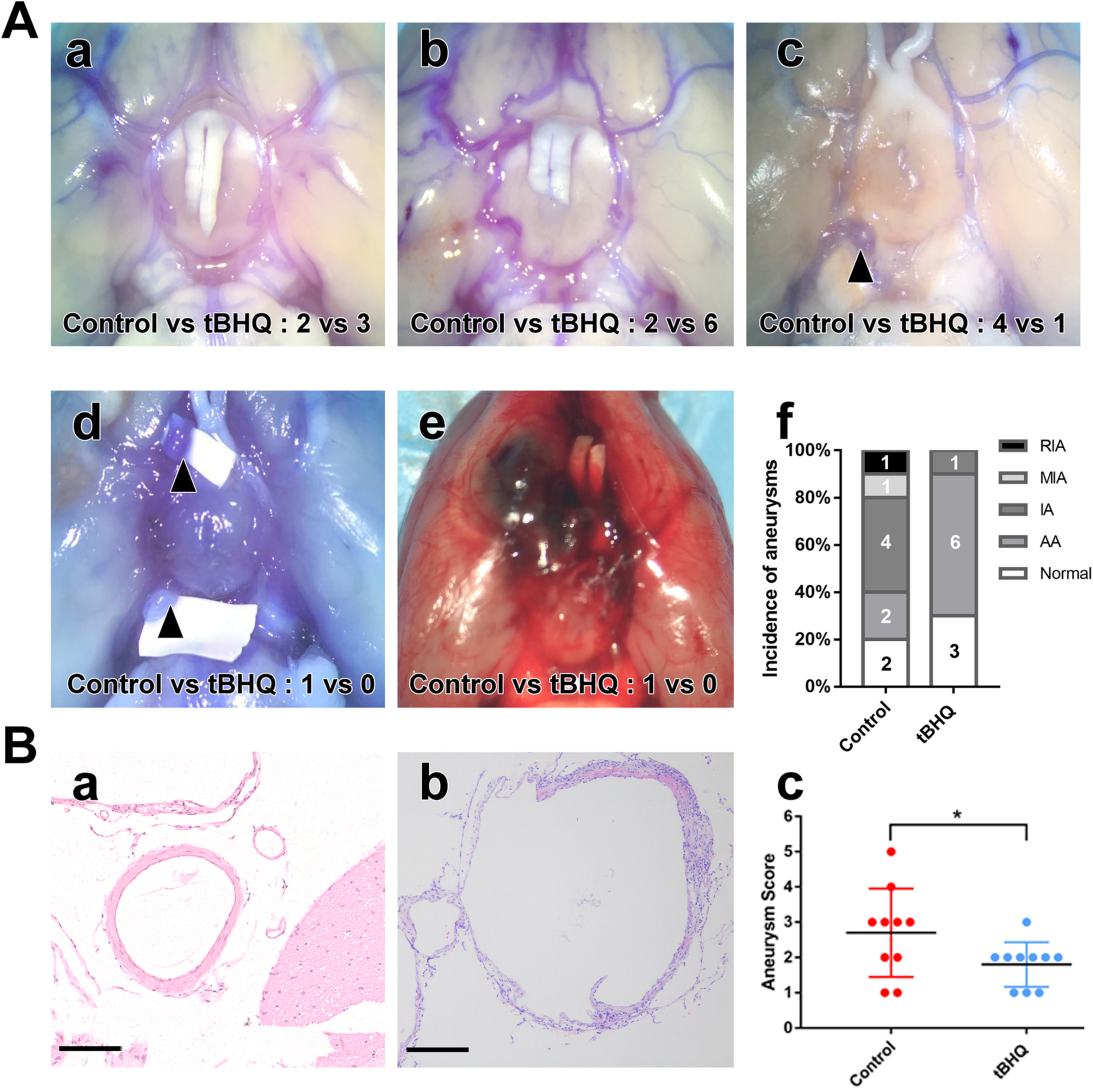
The effect of Nrf-2 activation on IA formation and progression. **a** Rate of aneurysm formation and rupture in the control and tBHQ treatment groups. (a) normal vessels, (b) aneurysmal artery (AA), (c) aneurysm (IA), (d) multiple aneurysms (MIA), (e) ruptured aneurysm (MIA), (f) quantitative analysis of (a) through (e) in the control and tBHQ treatment groups (*N* = intracranial aneurysms amounts under different condition). **b** (a) HE staining of normal cerebral artery (bar = 50 μM), (b) HE staining of IA (scale bar = 50 μM), (c) aneurysmal scores of the control and tBHQ treatment groups. Aneurysmal scores in the control group were much higher than that in the tBHQ group. (a) through (e). A corresponding to aneurysmal score of 1 to 5.*p < 0.05 compared with the control group

We first assessed the effect of Nrf-2 activation on IA formation and progression in a rat model. Figure 1B (a, b) HE staining show vessel wall weakness. Fig 1B shows the degradation of vessel wall and myointimal hyperplasia which are characteristics of unruptured intracranial aneurysm wall. Compared with normal cerebral artery, thinning of tunica media is more significant in the experimental group. Figure 1A (a) through (e) show normal cerebral arteries, aneurysmal arteries, an aneurysm, multiple aneurysms, and a ruptured aneurysm, respectively. The aneurysms are more likely to occur at major branching brain arteries. We found the rat aneurysms located at bifurcation of Willis’ circle and anterior circulation. There were seven and one aneurysms formed in the control (one middle cerebral artery, five anterior circulation, and one posterior circulation) and tBHQ (one anterior circulation) groups, respectively. These five types of arteries corresponded to different scores. The aneurysmal score of the tBHQ group was much lower than in the control group, suggesting that tBHQ can decrease the incidence of aneurysm and rate of rupture. There was a significant difference in aneurysm incidence between the control and tBHQ groups. The Nrf-2 agonist tBHQ significantly inhibited IA formation and progression in vivo.

### Downregulation and nuclear translocation of Nrf-2 in human IA specimens

**Fig. 2:**
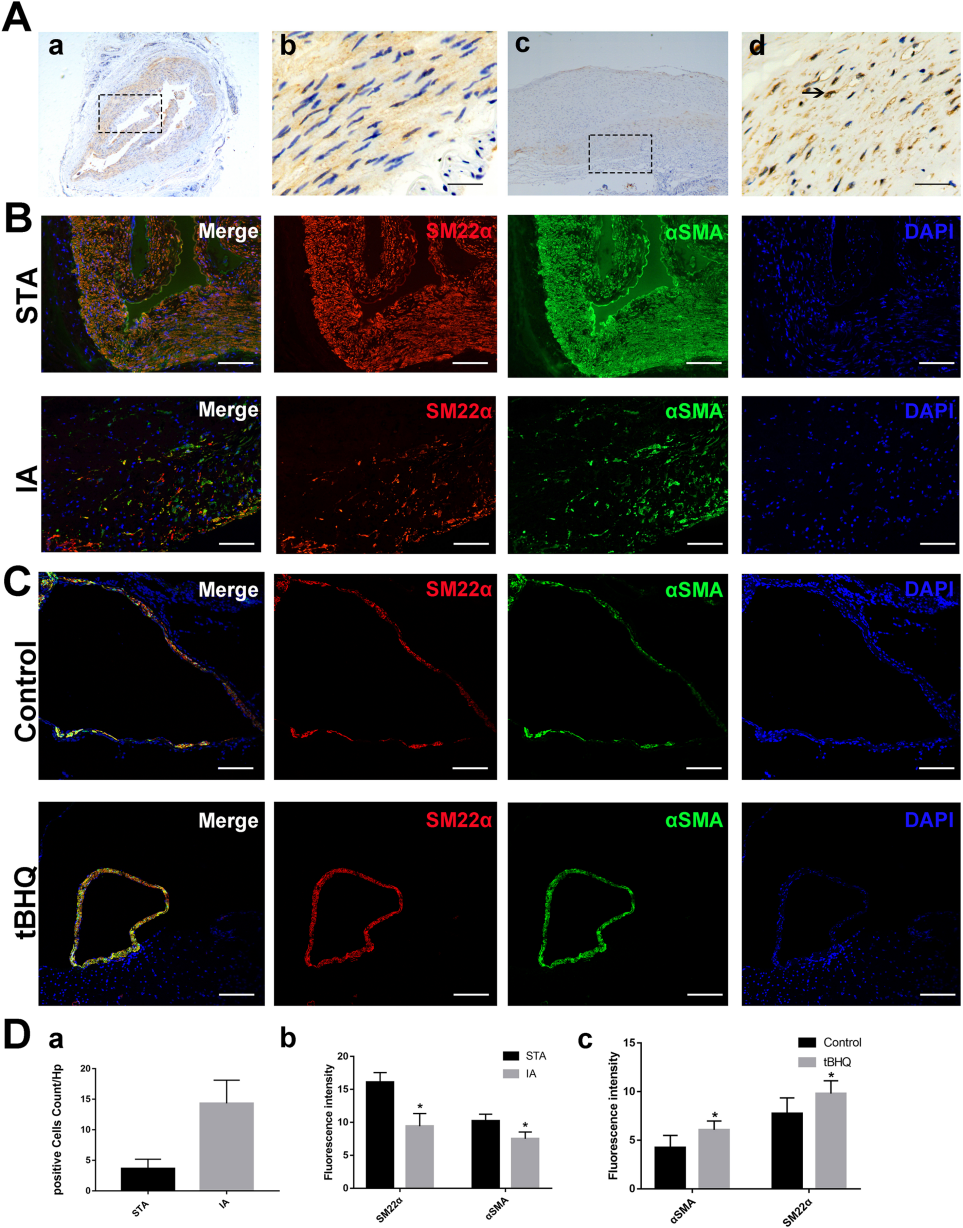
**a** Nrf-2 expression and localization in human STA (a, b) and IA (c, d). The decreased Nrf-2 expression and Nrf-2 nuclear translocation can be observed in magnified images (b, d) (scale bar = 10 μM). **b** Expression levels of VSMC-specific markers SM22α (red)/αSMA (green) and DAPI (blue) in human STA and IA (scale bar = 50 μM). The decline of VSMC-specific markers was detected in IA. **c** Representative pictures of the control and tBHQ groups for SM22α (red)/αSMA (green) and DAPI (blue) in immunofluorescence staining (scale bar = 50 μM). Photomicrographs show the increase of SM22α/αSMA in the tBHQ treatment group compared with the control. **d** Positive cells (cells with Nrf-2 translocation) numbers in **A** were analyzed (*N* = 3). Fluorescence intensity of specific VSMC markers in the STA/IA and control/tBHQ groups (*N* = 3). *p < 0.05 compared with the control group. STA, superficial temporal artery; IA, intracranial aneurysm; tBHQ, tert-butylhydroquinone

DAB staining demonstrated decreased Nrf-2 expression in IAs compared with STAs. Furthermore, Nrf-2 expression overlapped with the nucleus position in IA, indicating that more Nrf-2 translocated into the nucleus in IAs than STAs (Fig. 2A).

### VSMCs switched to the synthetic phenotype in human IA tissues

Based on previous studies, we chose SM22α and αSMA as markers of contractile phenotype VSMCs and found that levels of both were decreased in the synthetic phenotype. In STA, high immunofluorescent signals of SM22α/αSMA were detected in VSMCs with normal cellular morphology (Fig. 2B). However, SM22α and αSMA signals obviously declined in VSMCs in aneurysm walls (Fig. 2B), suggesting that VSMCs in IA walls switched to the synthetic phenotype.

### Nrf-2 agonist tBHQ can help VSMCs maintain a contractile phenotype

As mentioned above, the Nrf-2 activator tBHQ can reduce IA formation and inhibit progression in a rat IA model. SM22α and αSMA expression in aneurysms we developed in the control and tBHQ groups were analyzed. Aneurysms in the tBHQ group were smaller in diameter and had a thicker wall, a higher proportion of VSMCs, and greater SM22α/αSMA expression (Fig. 2C). The results indicated that tBHQ prevented VSMCs from decreasing in number and changing to the synthetic phenotype.

### H_2_O_2_ caused oxidative damage in VSMCs

**Fig. 3:**
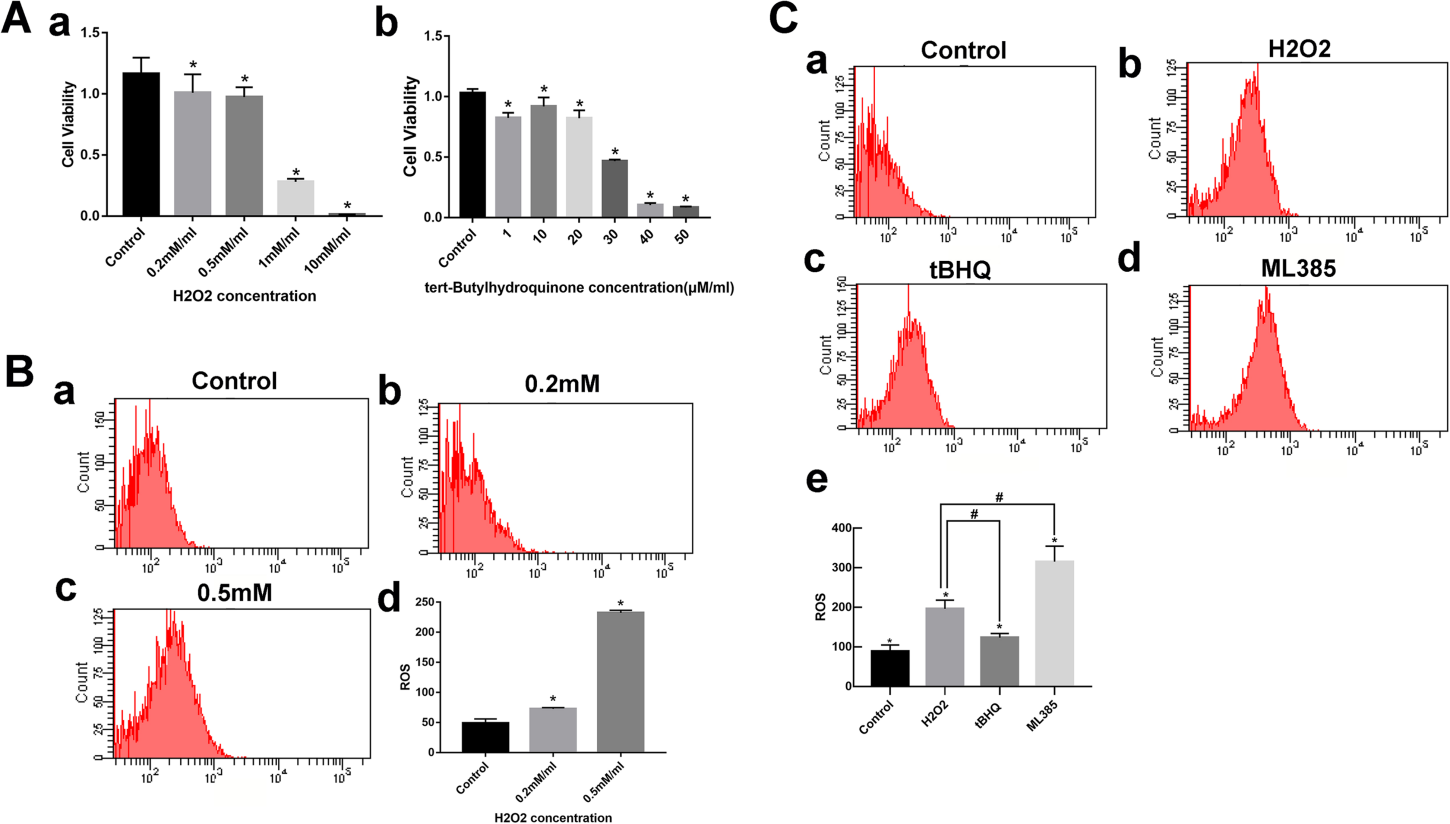
**a** Cell viability assays. (a) Viability changes of VSMCs treated with different concentrations of H_2_O_2_. Data are the means ± standard errors of the mean. *p < 0.05 compared with the control group. (b) Viability changes of VSMCs treated with different concentrations of tBHQ. Data are the means ± standard errors of the mean. *p < 0.05 compared with the control group. **b** Intracellular ROS in the control and H_2_O_2_ groups. Data are the means ± standard errors of the mean. *p < 0.05 compared with the control group. **c** Effect of Nrf-2 activator and inhibitor on H_2_O_2_ induced intracellular ROS levels. (a) control, (b) H_2_O_2_, (c) tBHQ, (d) ML 385. Data are the means ± standard errors of the mean. DCFH-DA lobe was used to measure levels of intracellular levels of ROS. *p < 0.05 compared with the control group; ^#^*p* < 0.05 compared with the H_2_O_2_ group

We initially intended to develop a VSMC oxidative damage model by incubation with H_2_O_2_ in vitro. ROS upregulation is known to cause oxidative damage. Cell viability was detected by CCK-8 assays, and intracellular ROS levels were by measured by flow cytometry. As shown in Fig. 3A (a) and Fig. 3B, H_2_O_2_ (0.5 mM) induced obvious intracellular ROS accumulation but had little influence on cell viability (*p* < 0.05). This concentration was therefore used in subsequent experiments.

### Nrf-2 activator tBHQ and Nrf-2 inhibitor ML 385 decreased and increased cellular ROS levels caused by H_2_O_2_, respectively

To access the protective effect of tBHQ on H_2_O_2_-induced cytotoxicity, VSMCs were treated with different tBHQ concentrations. At 20 μM/mL, tBHQ did not affect VSMC survival rate (Fig. 3A (b), *p* < 0.05). ML 385 treatment was performed as described by Biswal et al. [13]. Figure  3C shows H_2_O_2_ (0.5 mM) increased ROS levels yielding a twofold higher fluorescence intensity compared with the control group, but this was significantly reduced by tBHQ (*p* < 0.05). In contrast, intracellular ROS in the ML 385 group increased threefold compared to the H_2_O_2_ treatment group (*p* < 0.05). These results suggest that activation or inhibition of Nrf-2 could affect intracellular oxidative status.

### Nrf-2 inhibition contributed to VSMC proliferation and migration

**Fig. 4:**
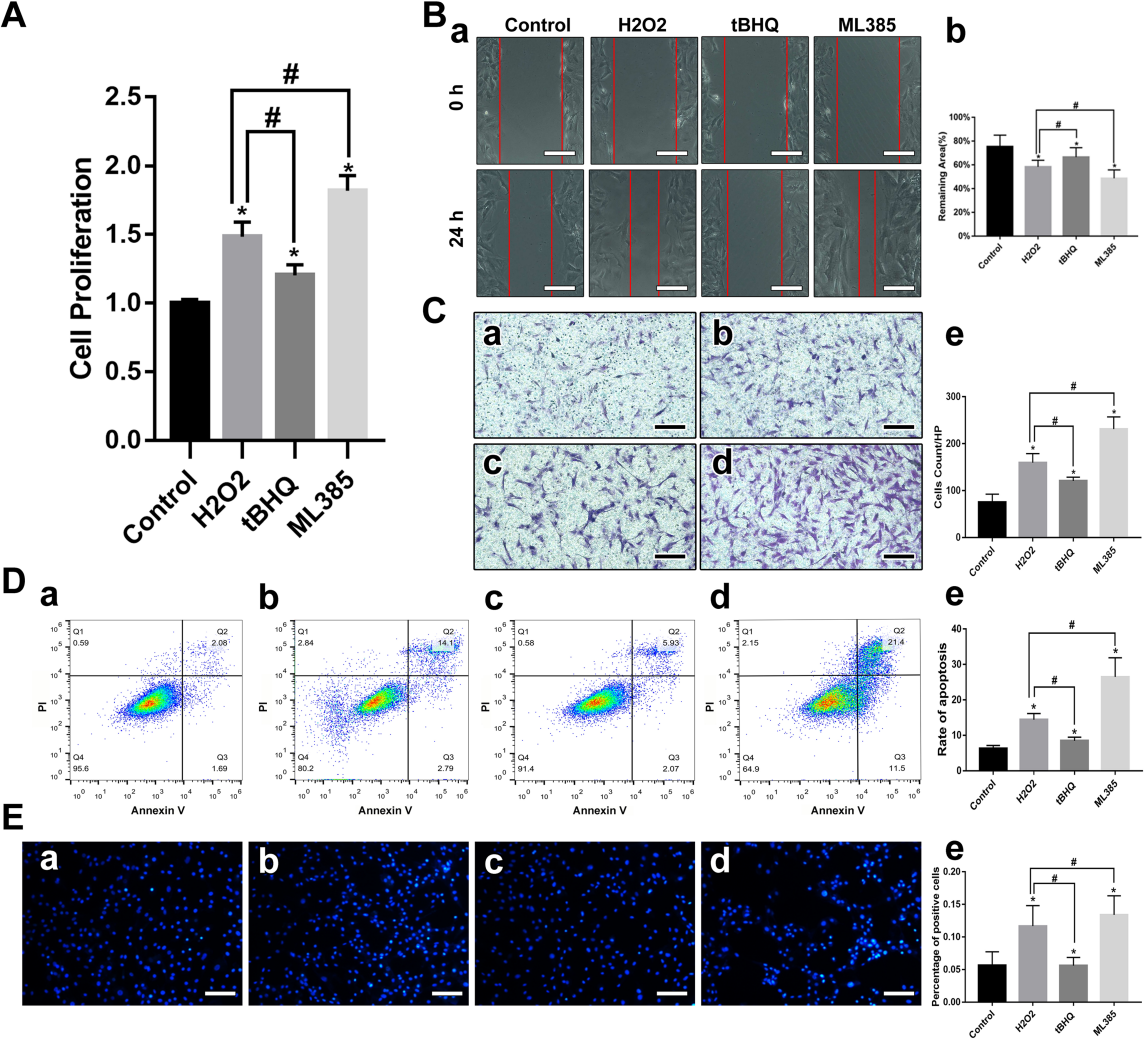
Nrf-2 signal pathway modulates VSMC functions and apoptosis rate. **a** Cell proliferation assays. Data are the means ± standard errors of the mean. *p < 0.05 compared with the control group; ^#^*p* < 0.05 compared with the H_2_O_2_ group. **b** Wound healing assays, representative images of HUVEC migration in the control, H_2_O_2_, tBHQ, and ML 385 groups in the scratch assay (scale bar = 50 μM). Data are the means ± standard errors of the mean. *p < 0.05 compared with the control group; ^#^*p* < 0.05 compared with the H_2_O_2_ group. **c** Transwell assays, representative images of invasive cells in the lower chamber stained with crystal violet (scale bar = 50 μM). (a) control, (b) H_2_O_2_, (c) tBHQ, (d) ML 385. Data are the means ± standard errors of the mean. *p < 0.05 compared with the control group; ^#^*p* < 0.05 compared with the H_2_O_2_ group. **d** Apoptosis rates as measured by flow cytometry. The Dead Cell Apoptosis Kit with Annexin V Alexa & propidium iodide (PI) was used to measure the apoptosis rate. (a) control, (b) H_2_O_2_, (c) tBHQ, (d) ML 385. Data are the means ± standard errors of the mean. *p < 0.05 compared with the control group; ^#^*p* < 0.05 compared with the H_2_O_2_ group. **e** Apoptosis rates analyzed by Hoechst staining. (a) control, (b) H_2_O_2_, (c) tBHQ, (d) ML 385. Data are the means ± standard errors of the mean. *p < 0.05 compared with the control group; ^#^*p* < 0.05 compared with the H_2_O_2_ group

Proliferation and migration abilities are different in the two distinct smooth muscle cell phenotypes [14]. Generally, synthetic VSMCs exhibit higher growth and migratory activity than contractile VSMCs [15]. We assessed cell viability after 12 h of H_2_O_2_ treatment with or without Nrf-2 activation. We found VSMCs oxidized by H_2_O_2_ exhibited higher proliferation ability, while tBHQ attenuated the H_2_O_2_-induced growth rate increase. Conversely, ML 385 pretreatment exacerbated this effect (Fig. 4A, *p* < 0.05). VSMC motility was analyzed by wound healing and transwell assays. The results (Fig. 4B and C) demonstrated that oxidative damage could enhance VSMC migration, and ML 385 treatment promoted migratory activity compared with the H_2_O_2_ group. A significant inhibition of migration was observed when VSMCs were incubated with tBHQ (*p* < 0.05). These results indicate that Nrf-2 could modulate VSMC proliferation and migration by regulating cellular oxidative stress.

### Effect of tBHQ and ML 385 on H_2_O_2_-induced vascular smooth muscle cells apoptosis

Oxidative stress leads to apoptosis. Hoechst staining and flow cytometry were performed to examine the apoptosis rate. As predicted, the VSMC apoptosis rate increased significantly after treatment with H_2_O_2_. Next, we detected the effect of Nrf-2 signaling on H_2_O_2_-induced VSMC apoptosis. Consistent with the effect of Nrf-2 on oxidative stress, tBHQ suppressed apoptosis, while ML 385 promoted apoptosis by inhibiting Nrf-2 (Fig. 4D and E, *p* < 0.05).

### Nrf-2 activation improves VSMC-specific gene expression

**Fig. 5:**
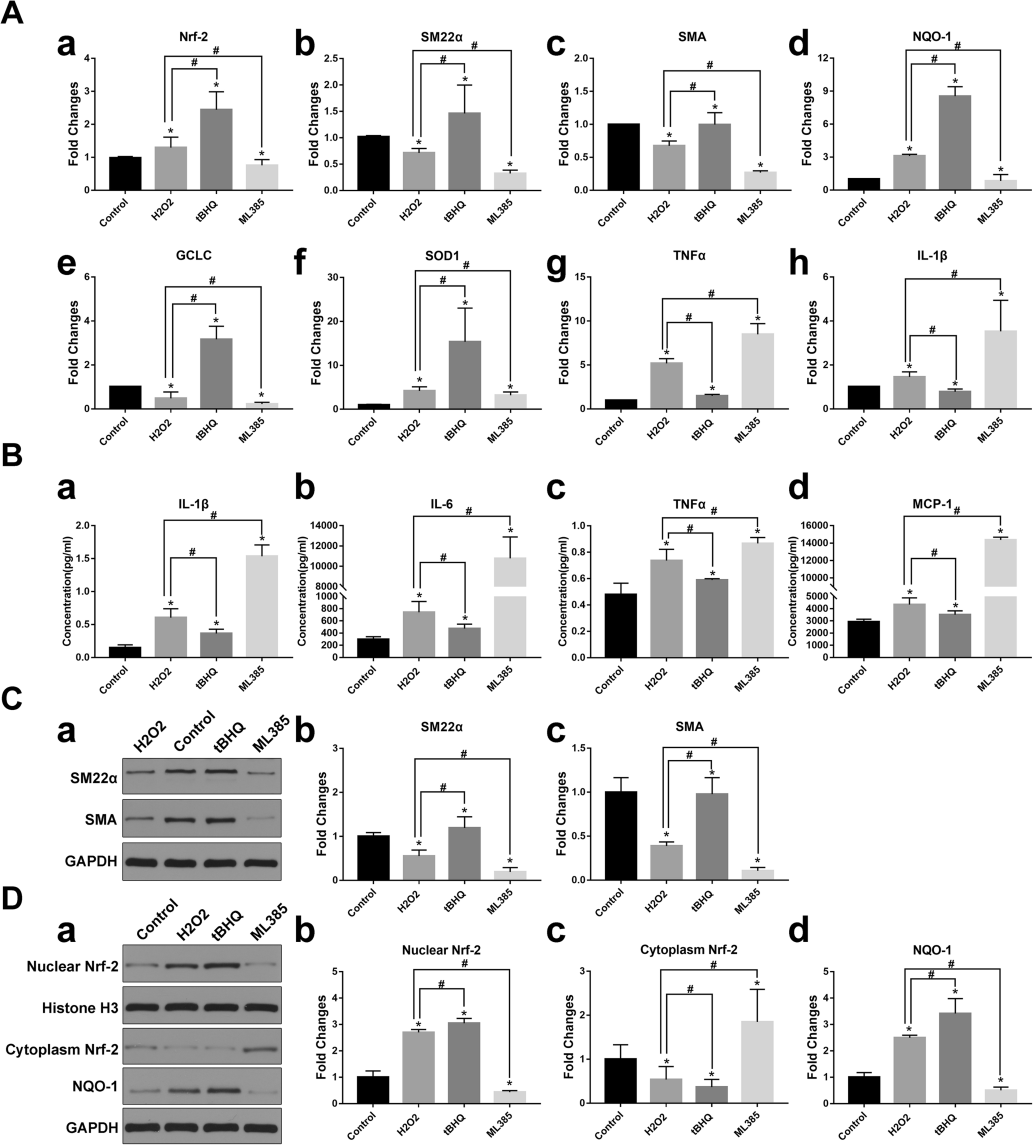
Effects of Nrf-2 signaling on VSMC-specific markers, antioxidant enzymes, and inflammatory cytokines. Representative images of experiments performed in triplicate are presented. **a** Fold changes of Nrf-2, downstream antioxidant enzymes, and inflammatory cytokines mRNA levels. Data are the means ± standard errors of the mean. *p < 0.05 compared with the control group; ^#^*p* < 0.05 compared with the H_2_O_2_ group. **b** Inflammatory cytokines analyzed by MILLIPLEX MAP Rat CVD Panel. Data are the means ± standard errors of the mean. *p < 0.05 compared with the control group; ^#^*p* < 0.05 compared with the H_2_O_2_ group. **c** Western blot analysis of VSMC specific markers SM22α, and αSMA. Data are the means ± standard errors of the mean. *p < 0.05 compared with the control group; ^#^*p* < 0.05 compared with the H_2_O_2_ group **d** Western blot analysis of Nrf-2 in the cytoplasm and nuclear fractions, antioxidant enzyme NQO-1, Data are the means ± standard errors of the mean. *p < 0.05 compared with the control group; #*p* < 0.05 compared with the H_2_O_2_ group

Several studies have described how oxidative stress can induce VSMCs to switch from the contractile to synthetic type. However, the role of Nrf-2, which is one of the most important antioxidant molecules in phenotypic modulation, is still unclear. Thus, qRT-PCR and western blot were performed to investigate the effect of Nrf-2 on the expression of VSMC-specific markers. To investigate whether tBHQ activates Nrf-2 or ML 385 inhibits Nrf-2, we analyzed Nrf-2 levels in the cytoplasm and nucleus. As shown in Fig. 5D, cytoplasmic Nrf-2 decreased and nuclear Nrf-2 increased after tBHQ treatment (*p* < 0.05). Conversely, ML 385 inhibited Nrf-2 nuclear translocation (*p* < 0.05). SM22α and αSMA mRNA and protein levels decreased in H_2_O_2_-incubated VSMCs. The inhibition of SM22α and αSMA was largely diminished in VSMCs pretreated with tBHQ, which was consistent with tBHQ-induced Nrf-2 nuclear translocation, whereas the Nrf-2 inhibitor ML 385 further exacerbated the decrease in both markers (Fig. 5A (a–c), Fig. 5C, *p* < 0.05).

### Impact of Nrf-2 activation on oxidative redox balance and inflammation

Nrf-2 is an important endogenous antioxidant defense molecule. Many enzymes downstream of the Nrf-2 pathway are primary components of the antioxidant system, also known as phase II detoxifying enzymes. The antioxidant enzymes examined by qRT-PCR and western blot included NQO-1, glutamate-cysteine ligase catalytic subunit (GCLC), and superoxide dismutase 1 (SOD 1). Inflammatory cytokines and oxidative stress are closely related. qRT-PCR and MILLIPLEX MAP were performed to detect inflammatory cytokines including IL-1b, IL-6, MCP-1, and TNF-a. Figure 5A and B shows that tBHQ increased antioxidant enzyme gene expression and downregulated cytokine levels by activating Nrf-2 (*p* < 0.05). ML 385 aggravated oxidative damage by upregulating inflammatory cytokines and inhibited the transcription of genes encoding antioxidant enzymes (*p* < 0.05).

## Discussion

The mechanism of IA development is still unclear. Many pathological factors like oxidative stress, inflammation, dysregulated hemodynamics, and extracellular matrix degeneration contribute to their formation and progression [16]. The interaction between oxidative stress and inflammation is a key mechanism of endothelial dysfunction and arterial damage. A role of VSMCs phenotype switching has been described in IAs, atherosclerosis, and aortic aneurysms. Oxidative stress can accelerate inflammation directly and indirectly, which further increases oxidative stress. This can induce phenotypically switched VSMCs to stimulate pro-inflammatory processes, extracellular matrix remodeling, and mural cells apoptosis, all of which eventually contribute to IA formation and rupture. Numerous lines of evidence indicate that VSMC phenotypic modulation is associated with IA formation, growth, and rupture [17–19].

Nrf-2, a basic leucine zipper transcription factor, plays a critical role in modulating cellular redox balance by promoting the transcription of detoxifying and antioxidant genes. Under oxidative conditions, Nrf-2 separates from Keap1 in the cytoplasm and translocates to the nucleus, which leads to the transcription of phase II detoxification enzymes and anti-inflammatory proteins. Researchers previously demonstrated that VSMCs phenotypes are influenced by inhibiting ROS generation, and this is important for maintaining vascular homeostasis [20]. Other findings also suggest that Nrf-2 could affect VSMC functions by reducing oxidative stress [21, 22]. We hypothesized that Nrf-2 pathway activation can prevent VSMCs from switching to the synthetic type by modulating oxidative stress and exerting protective effects against IA development.

We first examined the role of Nrf-2 pathway activation in vivo. An elastase-induced rat IA model was developed, and arteries were divided into five different types: normal cerebral arteries, aneurysmal arteries, aneurysms, multiple aneurysms, and ruptured aneurysms. After 30 days of treatment with Nrf-2 agonist tBHQ, there were lower aneurysmal scores and less IA formation and rupture compared with the control (Fig. 1). This indicates that Nrf-2 activation can suppress IA formation and progression. Consistent with our data, Zeng et al. reported that increased Nrf-2 levels help inhibit acute aortic dissection formation, which involves a pathological mechanism similar to IA [23]. In the presence of stimulating factors, Nrf-2 translocates into the nucleus and promotes downstream target genes expression by binding to the antioxidant response element. Our immunohistochemical results revealed stabilized Nrf-2 distributed in the cytoplasm of STAs, while downregulation and nuclear translocation were found in aneurysm walls (Fig. 2A). Nakajima et al. detected VSMCs phenotypic modulation in human aneurysmal walls and concluded that this process was related to aneurysm remodeling and rupture [24]. We found VSMC morphological changes, decreased SM22α/αSMA expression, and loss of mural VSMCs in IA, all of which are characteristic of IA walls (Fig. 2B). In the rat IA model, SM22α/αSMA was upregulated and aneurysm diameter was smaller after tBHQ treatment (Fig. 2C). These results suggest that Nrf-2 activation inhibits IA development by modulating VSMCs phenotype.

To investigate how Nrf-2 contributes to VSMC phenotypic switching, we examined the effect of Nrf-2 activation or inhibition on VSMC phenotype changes and functions in vitro. H_2_O_2_ can promote ROS generation, which is a central molecular component of oxidative stress and is widely used to induce cellular damage [25, 26]. We selected tBHQ as a Nrf-2 activator and ML 385 as Nrf-2 inhibitor, since they are well-studied and specific for Nrf-2 [13, 27, 28]. We observed that tBHQ decreased ROS generation while ML 385 promoted significant ROS accumulation (Fig. 3C). The results of cell proliferation and migration tests revealed that Nrf-2 activation exerts a negative effect on proliferative and migratory abilities. Two different methods confirmed that Nrf-2 activation decreased apoptosis (Fig. 4). Therefore, Nrf-2, which has regulatory roles in VSMC proliferation, migration, and apoptosis, is also associated with vascular remodeling that leads to IA formation and progression. We demonstrated that VSMCs switched to the synthetic phenotype after oxidative treatment, but while tBHQ helped, VSMCs maintain contractile phenotype according to qRT-PCR and western blot results. Conversely, ML 385 profoundly suppressed VSMC-specific marker expression (Fig. 5). The underlying mechanism may involve upregulation of antioxidant enzymes, decreased pro-inflammatory cytokine expression, and less intracellular ROS accumulation (Fig. 3, Fig. 5). When SM22α/αSMA levels are decreased, VSMCs develop increased capacities for proliferation, migration, and secretion of pro-remodeling factors and pro-inflammatory cytokines [29, 30]. VSMCs undergoing phenotypic switching can also aggravate oxidative stress and inflammation. These results are consistent with the functional changes described above and suggest that Nrf-2 modulates VSMC phenotype switches by affecting antioxidant and anti-inflammatory protein expression.

## Conclusion

**Fig. 6:**
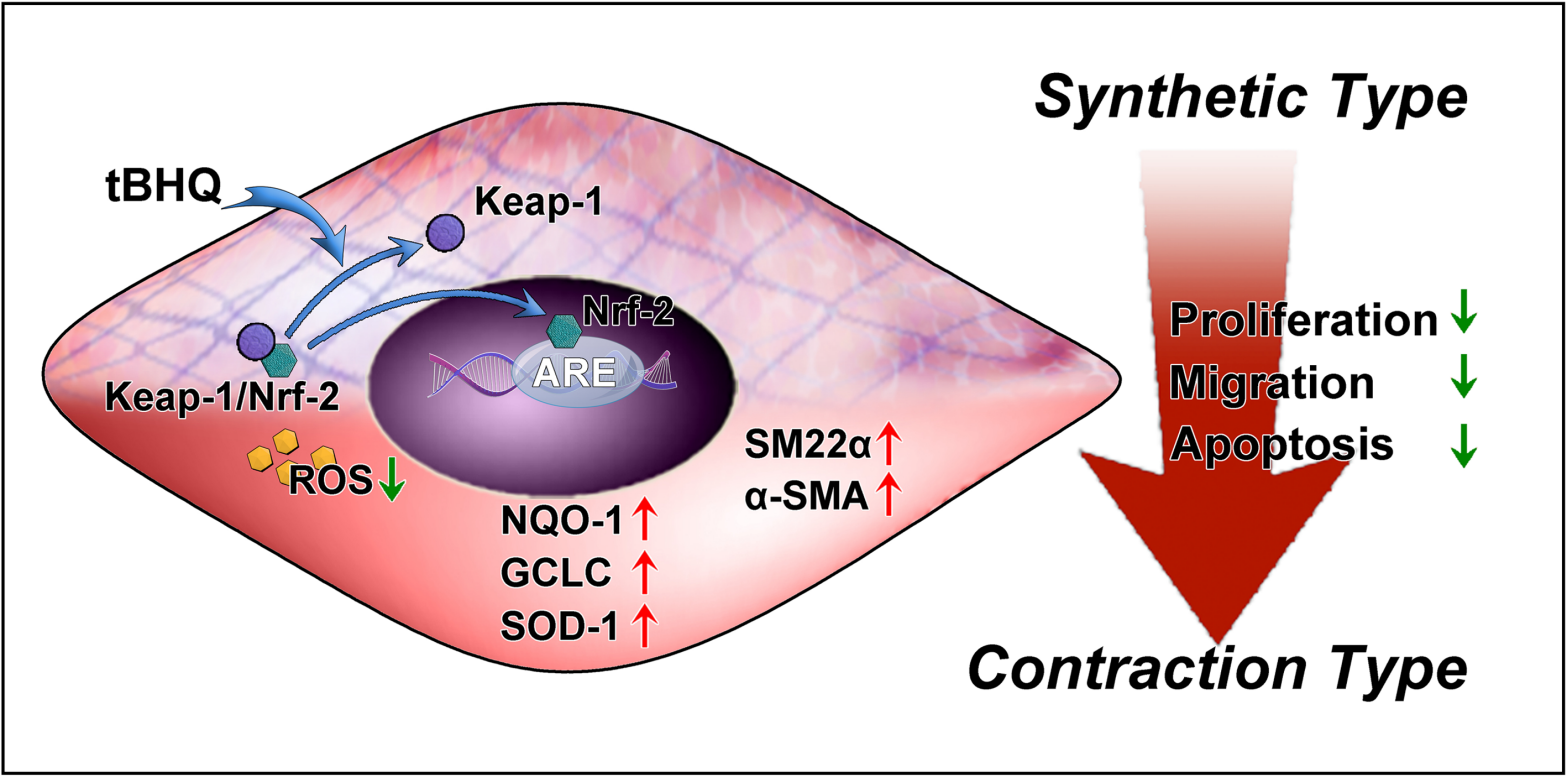
Schematic illustrating how Nrf-2 activation regulates phenotypic modulation. Under oxidative-stimulating conditions, Nrf-2 is activated and translocates into the nucleus. Nrf-2 signal pathway activation promotes expression of VSMC-specific markers SM22α and αSMA. The underlying mechanism may be that Nrf-2 upregulates expression levels of its downstream antioxidant enzymes and inhibits pro-inflammatory cytokines

In summary, our results provide novel evidence that Nrf-2 signaling can inhibit IA formation and progression by regulating VSMC phenotypes. The Nrf-2 agonist tBHQ decreases the rates of IA formation and rupture and increases VSMC contractile gene expression by promoting Nrf-2 nuclear translocation (Fig. 6). The Nrf-2 antagonist ML 385 reverses this effect. Although prior studies have found a close relationship between VSMC phenotype switching and IA formation and rupture, none have assessed the role of the Nrf-2 signal pathway. Our findings provide a theoretical basis for research into novel pharmacologic therapies.

## Data Availability

Data generated and analyzed as part of this study are included in the manuscript or are available upon request from the corresponding author.

## Abbreviations

ECM: Extracellular matrix
HE: Hematoxylin and eosin
IA: Intracranial aneurysm
MHC: Myosin heavy chain
NQO-1: NAD(P)H quinone dehydrogenase 1
Nrf-2: Nuclear factor erythroid 2-related factor 2
PCR: Polymerase chain reaction
ROS: Reactive oxygen species
SM22α: Smooth muscle 22 alpha
SM-α-actin: Smooth muscle alpha actin
tBHQ: Tert-butylhydroquinone
VSMC: Vascular smooth muscle cell
